# Vasospasm in the First Septal Perforator Branch and Late High-Grade Atrioventricular Block Following Successful Primary Percutaneous Coronary Intervention for the Proximal Left Anterior Descending Coronary Artery: A Case Report

**DOI:** 10.7759/cureus.39172

**Published:** 2023-05-18

**Authors:** Koji Takahashi, Masafumi Takemoto, Tomoki Sakaue, Shuntaro Ikeda, Takafumi Okura

**Affiliations:** 1 Department of Cardiology, Yawatahama City General Hospital, Yawatahama, JPN; 2 Department of Medical Engineering, Yawatahama City General Hospital, Yawatahama, JPN; 3 Department of Community Emergency Medicine, Ehime University Graduate School of Medicine, Matsuyama, JPN

**Keywords:** primary percutaneous coronary intervention, methylergometrine, left anterior descending coronary artery, implantable loop recorder, first septal perforator branch, coronary vasospasm, advanced atrioventricular block

## Abstract

We present a case of a high-degree advanced atrioventricular block (AVB), which occurred 24 hours after successful primary percutaneous coronary intervention (PCI) in the proximal left anterior descending coronary artery (LAD), the culprit of ST-segment elevation myocardial infarction (STEMI). The methylergometrine provocation test for coronary vasospasms, which was performed on the eighth hospital day, revealed transient total occlusion of the first septal perforator branch. After prescribing a calcium channel blocker to the patient, AVB did not recur for three years, as confirmed using an implantable loop recorder (ILR). In this patient, delayed high-grade AVB following primary PCI in the proximal LAD might be caused by the spasm of the first septal perforator branch. Documented cases of spasms in this branch are rare.

## Introduction

High-grade atrioventricular block (AVB), a complication of ST-segment elevation myocardial infarction (STEMI), remains associated with higher rates of permanent pacemaker implantation and in-hospital mortality even in the current era of prompt reperfusion therapy. Compared with patients with STEMI due to right coronary artery occlusion, this is particularly true in patients with STEMI due to left anterior descending coronary artery (LAD) occlusion [1-3].

The first septal perforator branch supplies blood to the atrioventricular node and bundle of His in 50% of the population [4]. Reports of complete heart block due to the occlusion of the first septal perforator branch are increasing [5,6]. Here, we present a case of an advanced AVB that occurred 24 hours after successful primary percutaneous coronary intervention (PCI) in the proximal LAD, the culprit vessel of STEMI. Coronary provocative testing with intracoronary methylergometrine, which was performed on the eighth hospital day, revealed transient total occlusion of the first septal perforator branch, but not the LAD trunk. In our patient, delayed high-grade AVB following primary PCI in the proximal LAD might have been caused by a spasm of the first septal perforator branch. Documented cases of spasms in this branch are rare [7,8].

## Case presentation

A 56-year-old Japanese male was admitted to our hospital because of severe chest squeezing associated with diaphoresis that lasted for 10 hours. Seven days prior, he experienced mild squeezing in the chest without associated symptoms, which occurred on effort several times a day. The patient had no history of smoking or alcohol consumption. His significant medical history findings were as follows: long-standing hypertension, renal transplantation for end-stage renal failure performed 25 years prior to presentation, cholecystectomy, thyroid tumor removal, and tonsillectomy. The patient was prescribed oral tacrolimus hydrate (2 mg once daily), mycophenolate mofetil (500 mg twice daily), methylprednisolone (2 mg once daily), combination granules of sulfamethoxazole/trimethoprim (1 g once daily), candesartan cilexetil (8 mg once daily), cilnidipine (10 mg once daily), and rabeprazole sodium (10 mg once daily) at another clinic.

In the emergency room, his vital signs were as follows: temperature, 36.9°C; pulse rate, 75 bpm; systemic blood pressure, 129/96 mm Hg; and oxygen saturation (on room air measured using a pulse oximeter), 97%. Physical examination findings were unremarkable. Blood test results indicated significant myocardial injury and moderately decreased kidney function (Table 1). In addition, blood tests for the risk of coronary artery disease revealed normal lipid profiles but slightly elevated glucose levels.

**Table 1:** Laboratory test values on admission hs-cTnI, high-sensitivity cardiac troponin I; BNP, brain natriuretic peptide; LDL, low-density lipoprotein; HDL, high-density lipoprotein; eGFR, estimated glomerular filtration rate

Blood test parameter	Result	Reference value
C-reactive protein (mg/dL)	0.04	≤0.7
Creatine kinase (U/L)	3,004	≤190
Creatine kinase-MB (U/L)	286	≤25
hs-cTnI (pg/mL)	>50,000	≤18.4
BNP (pg/mL)	103.4	≤18.4
LDL cholesterol (mg/dL)	96	≤140
HDL cholesterol (mg/dL)	62	35-70
Glucose (mg/dL)	133	Fasting: 70-109
Glycated hemoglobin A1c (%)	6.1	4.6-6.2
eGFR (mL/minute/1.73 m^2^)	50	90-120

**Figure 1:**
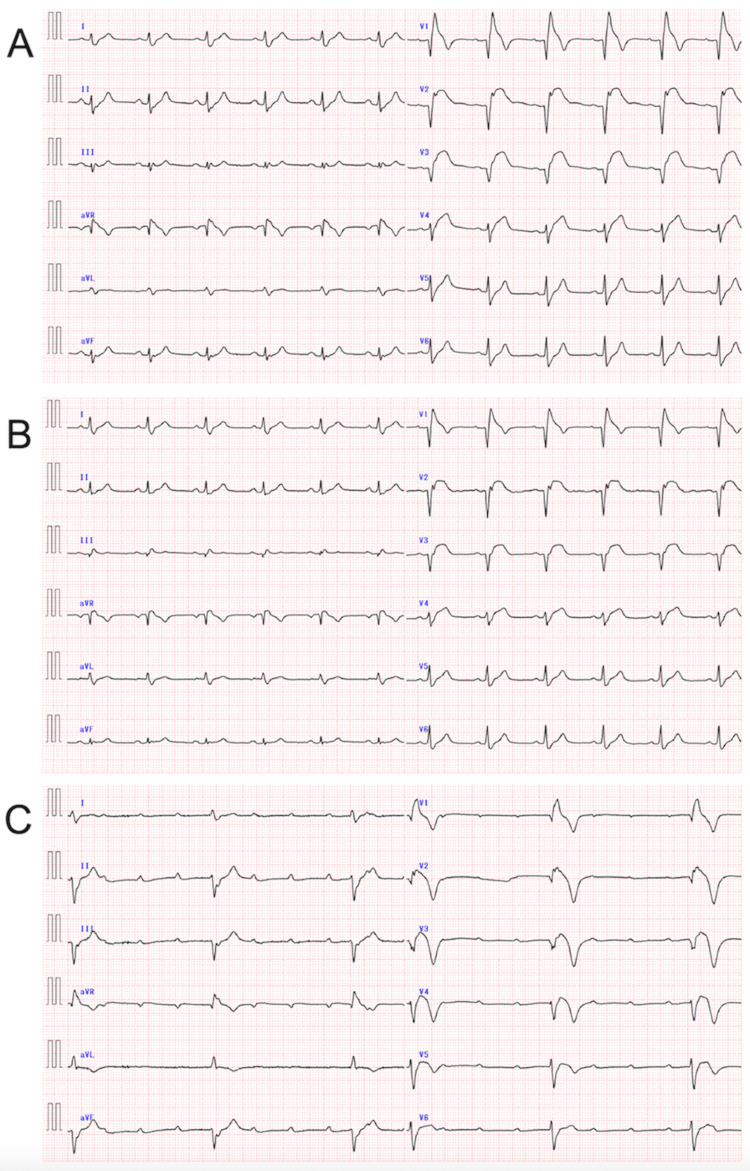
Electrocardiograms recorded on admission (A) and at eight hours (B) and 24 hours (C) after primary percutaneous coronary intervention in the proximal left anterior descending coronary artery A shows a sinus rhythm with complete right bundle branch block, Q-waves in leads V1 through V3, and ST-segment elevations of 0.6 mV maximum in leads I, aVL, and V1 through V4, concerning the anteroseptal wall ST-segment elevation myocardial infarction. B shows a decreasing trend in the degree of ST-segment elevations in leads I, aVL, and V1 through 4, as compared with that recorded on admission. C shows a complete atrioventricular block and junctional rhythm with a QRS rate of 28 beats/minute.

Chest radiography revealed no pulmonary congestion. An electrocardiogram (ECG) revealed anteroseptal wall STEMI with a complete right bundle branch block (Figure 1A). A standard echocardiogram showed severe hypokinesis to akinesis in the left ventricular anterior wall and anterior interventricular septum. Thus, the patient was diagnosed with STEMI.

**Figure 2:**
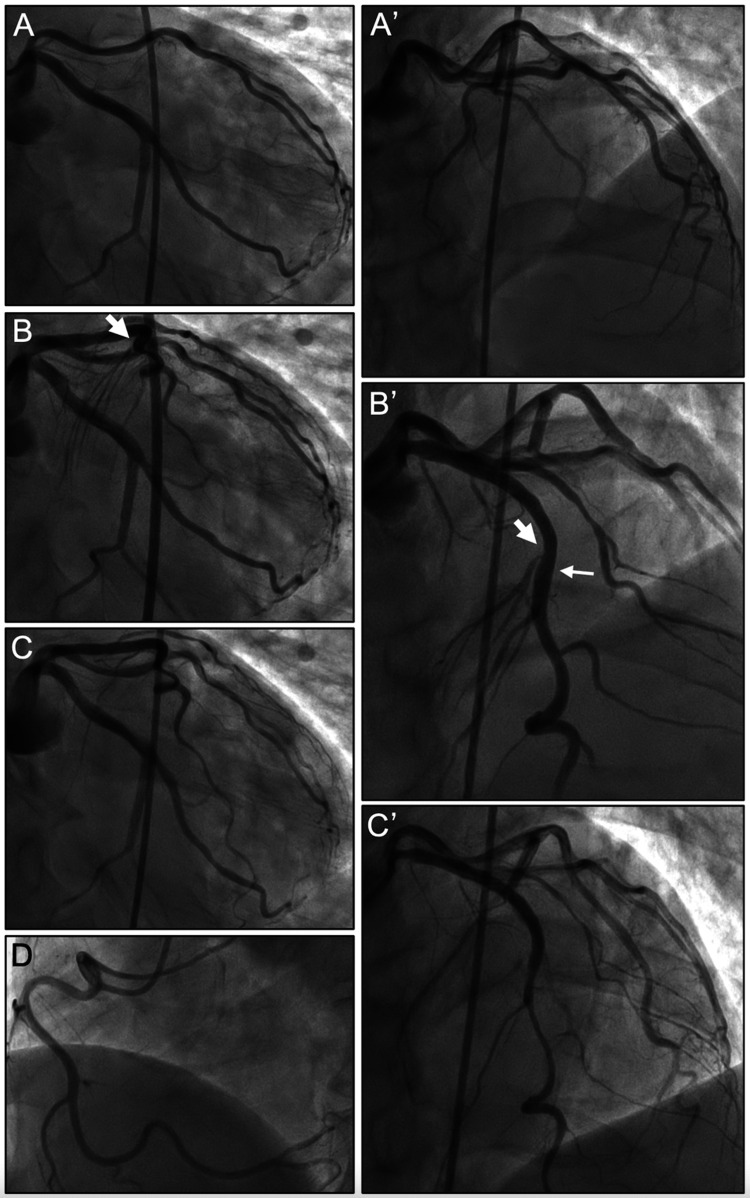
Coronary angiograms on admission and on the eighth hospital day The emergent coronary angiography performed on admission revealed subtotal occlusion with a thrombolysis in myocardial infarction grade 1 flow in the proximal LAD and no significant stenosis in the left circumflex coronary or right coronary artery (A, A’, and D). After thrombus aspiration therapy and coronary stent deployment, coronary angiography revealed no residual stenosis in the culprit lesion but 75% discrete stenosis at the origin of the first septal perforator branch, which takes off from the stent site of the LAD (B and B’). On the eighth hospital day, a provocative test for coronary vasospasms revealed total occlusion in the first septal perforator branch, not the LAD trunk, after 64 μg of methylergometrine maleate was injected into the left coronary artery over a period of three minutes (C and C’). The large arrows indicate severe stenosis in the first septal perforating branch. The small arrows indicate the distal sites of the stent implanted in the LAD. The images in A, B, and C represent the anteroposterior and 30° caudal views, those in A’, B’, and C’ represent the anteroposterior and 30° cranial views, and that in D represents the 50° left anterior oblique view. LAD, left anterior descending coronary artery

The patient continued to experience sustained chest pain. He received dual-loading antiplatelet therapy (aspirin, 324 mg; prasugrel hydrochloride, 20 mg) and underwent emergent coronary angiography, which revealed subtotal occlusion with thrombolysis in myocardial infarction (TIMI) grade 1 flow in the proximal LAD (Figure 2A, 2A’). Continuous intravenous infusion with nicorandil was started at 4 mg/minute. First, thrombus aspiration therapy, which was performed using a 7-French thrombus aspiration catheter, led to the withdrawal of a large amount of a dark-brown blood clot; therefore, a TIMI grade 3 flow was achieved. Subsequently, an intravascular ultrasonography-guided 2.75 × 24 mm everolimus-eluting platinum chromium coronary stent was deployed to the lesion and post-dilated with a 2.75 mm non-compliant balloon without residual stenosis. The intravenous nicorandil infusion was continued.

**Figure 3:**
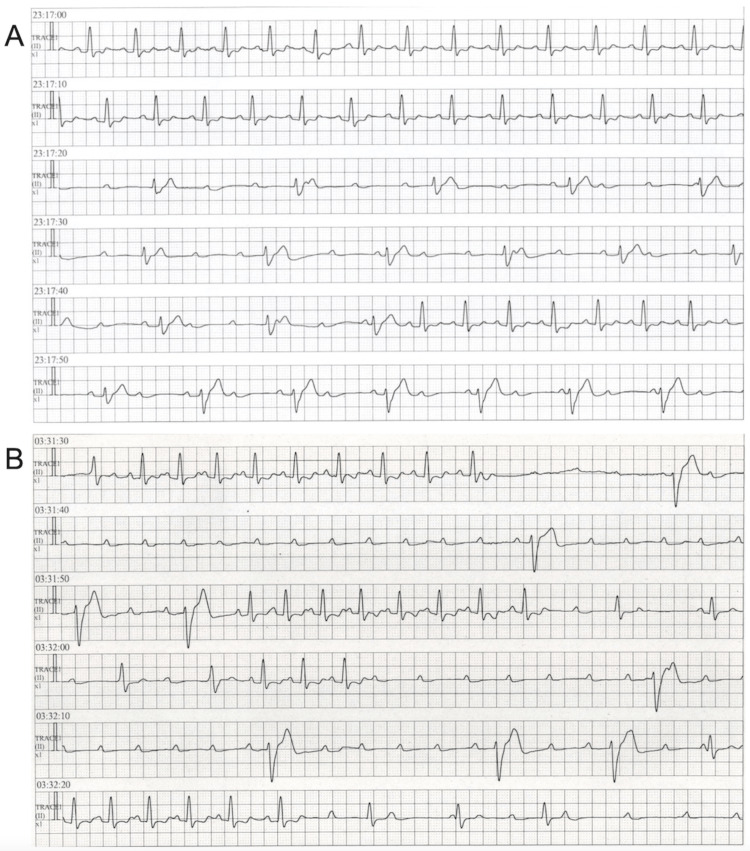
Rhythm strips of continuous electrocardiograms from bedside cardiac monitors recorded during two episodes of AVB at 11 p.m. (A) and 3 a.m. (B), approximately 24 hours after a primary percutaneous coronary intervention on the proximal left anterior descending coronary artery A and B show complete AVB with escape rhythm and advanced AVB with scattered idioventricular beats, respectively. Two AVBs developed abruptly without significant ST-segment deviation. The longest ventricular standstill lasted for approximately eight seconds. AVB, atrioventricular block

The patient was completely relieved of his symptoms after revascularization. On the second hospital day, cilnidipine was withheld because of hypotension, and nicorandil was switched from 96 mg daily (administered via continuous intravenous infusion) to 20 mg daily (administered orally). The peak creatine kinase level was 6,687 U/L. A follow-up ECG revealed a decreasing trend in the degree of ST-segment elevations in the anterior chest leads (Figure 1B). However, 24 hours after reperfusion, complete and advanced AVBs for approximately 40 minutes were documented at 11 p.m. and 3 a.m. (Figure 3). At these times, the patient became unconscious with mild generalized twitching, although chest pain was not observed. Consequently, a temporary pacing catheter was inserted.

On the eighth hospital day, nicorandil was discontinued, and the patient underwent follow-up coronary angiography, which revealed no interval changes in the stenting site and 75% discrete stenosis at the origin of the first septal perforator branch (Figure 2B, 2B’). Thus, a provocative test for coronary vasospasm was performed using methylergometrine maleate as the provocative stimulus [9]. A total of 64 μg of methylergometrine maleate was injected into the left coronary artery for over three minutes. Total occlusion in the first septal perforator branch, not the LAD trunk, without chest pain or ischemic ECG changes including AVB was induced one minute after completing the injection (Figure 2C, 2C’). We considered the provocation test findings to be positive; thus, 2 mg of isosorbide dinitrate was injected into the left coronary artery immediately, and the provoked vasospasm of the first septal perforator branch was resolved. Methylergometrine maleate was not injected into the right coronary artery because the ECG at the time of advanced AVB appearance did not reveal inferior ST-segment elevation, which ruled out the possibility that a spasm in the right coronary artery caused the AVB. Cardiac pacing was discontinued.

**Figure 4:**
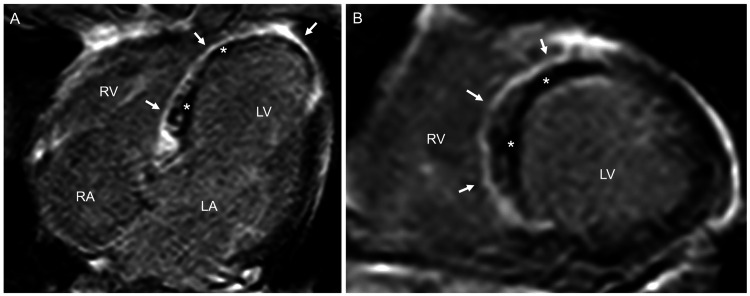
Delayed contrast-enhanced cardiac magnetic resonance images (A, four-chamber view; B, short-axis view) obtained on the ninth hospital day Images show a transmural hyperenhancement (arrows) in the whole area at risk of a myocardial infarction, which corresponded to the necrotic area and surrounded a dark core (asterisks) that reflected the microvascular obstruction. LA, left atrium; LV, left ventricle; RV, right ventricle; RA, right atrium

Left ventricular parameters, which were obtained on cine-balanced steady-state free precession images of cardiac magnetic resonance performed on the ninth hospital day, revealed a normal end-diastolic volume index of 76.2 mL/m^2^ and a reduced ejection fraction of 32.1% [10]. In addition, delayed contrast-enhanced cardiac magnetic resonance images revealed that the area perfused by the proximal LAD had almost no viability (Figure 4). Although there was significant stenosis at the bifurcation of the first septal branch, the lack of viability in the perfusion extent of this vessel led us to conclude that there was no indication for revascularization in this vessel; however, treatment to prevent spasm was warranted. Thus, the patient was switched from nicorandil (10 mg twice daily) to amlodipine besilate (2.5 mg twice daily). Furthermore, carvedilol was initiated at a dose of 2.5 mg twice daily to prevent left ventricular remodeling. The patient was discharged after subcutaneous implantation of an implantable loop recorder (ILR), Reveal LINQ (Medtronic Inc., Minneapolis, MN, USA), into the left parasternal region (45° to the sternum over the fourth intercostal space, 2 cm from the left edge of the sternum) for the early detection of AVB on the 10th hospital day. Three years later, AVB did not recur in the patient, as confirmed through ambulatory ECG monitoring using the ILR. Transthoracic echocardiography did not reveal a worsening left ventricular remodeling, with an ejection fraction of 45%.

## Discussion

In patients with STEMI due to LAD occlusion, a high-degree AVB develops even in the late convalescent phases [11]. Thus, it was difficult to differentiate between AVB caused by myocardial infarction per se or obstruction of the first septal perforator branch in this patient. Spontaneous or iatrogenic obstruction of the first septal perforator branch can usually lead to AVB [5,6,12-14]. In this patient, two episodes of transient AVB occurred around midnight during the acute phase of anterior STEMI [15]; coronary provocative testing with intracoronary methylergometrine revealed transient obstruction of the first septal perforator branch, although AVB was not detected during the tests. In addition, the patient never experienced an incidence for three years after the prescription of a calcium channel blocker; thus, delayed high-grade AVB following primary PCI in the proximal LAD might have been caused by the spasm of the first septal perforator branch.

A positive provocative test with high specificity and sensitivity for coronary artery spasms must induce severe vasoconstriction accompanied by chest pain (typical of the patient’s usual complaint) and ischemic ECG changes in response to the provocative stimulus, such as ergonovine and acetylcholine [9,16,17]. The test result is considered equivocal if the provocative stimulus does not induce all three components [9]. In this patient, chest pain or ischemic ECG changes, such as ST-segment shift and negative U-wave, did not occur in time with the total occlusion in the first septal perforator branch. However, cardiac magnetic resonance imaging findings revealed that the area perfused by the proximal LAD almost had no viability, which may hardly lead to chest pain, or ischemic ECG changes, when the first septal perforator branch was transiently occluded [8]. Thus, we considered the provocative test to be positive for vasospasm in this branch. In addition, we thought that a provoked total occlusion of the first septal perforator branch for a few minutes could induce right bundle branch block or left fascicular blocks but unlikely to induce AVB [7,18], although studies have revealed high rates of AVB occurrence due to long-term or permanent occlusion of that vessel in patients with myocardial infarction or hypertrophic cardiomyopathy who undergo alcohol septal ablation [6,12,13]. The timing of when to perform a provocation test for coronary vasospasm after primary PCI is debatable, although the test itself is safe [19].

The incidence of complete AVB, which is usually within the His-Purkinje system and related to the interruption of septal perfusion accompanied by extensive myocardial damage and significant left ventricular dysfunction, in patients with anterior STEMI was 0.9% [1]. Even with prompt reperfusion therapy, such as primary PCI limiting infarct size, complete AVB, although transient in most patients, remains associated with significantly higher in-hospital mortality [2]. In such cases, the rates of temporary and permanent pacing therapies during hospitalization are 31% and 18.2%, respectively. In addition, Gang et al. [11] reported that high-grade AVB was documented by ILR in 28 (10%) out of 292 patients with late convalescent phases of myocardial infarction and left ventricular dysfunction during a median follow-up of two years. We decided that continuous ECG monitoring using ILR was warranted in our patient who presented with transient advanced AVB and left ventricular systolic dysfunction following STEMI of the proximal LAD, although ILR cannot detect ischemic ECG changes and there is no evidence that an ILR-based diagnostic strategy reduces long-term mortality compared with a standard diagnostic assessment [20]. In our patient, ILR did not detect either AVB or ventricular tachyarrhythmia for three years.

## Conclusions

We present a case of advanced AVB that occurred 24 hours after successful PCI in the proximal LAD, the STEMI-related lesion. The methylergometrine provocation test for coronary vasospasm, which was performed in the subacute stage of STEMI, revealed transient total occlusion of the first septal perforator branch. After prescribing a calcium channel blocker to the patient, AVB did not recur for three years, as confirmed using ILR. Although delayed high-grade AVB following primary PCI in the proximal LAD is caused by STEMI per se, this bradyarrhythmia in our patient might have been caused by a spasm of the first septal perforator branch. Thus, a provocation test for coronary vasospasms might be warranted in this clinical setting, although the timing of when to perform the test after primary PCI is debatable. Moreover, continuous ECG monitoring using ILR might be useful in patients with transient advanced AVB and left ventricular systolic dysfunction following STEMI of the proximal LAD, as shown in our patient.
